# Intergenerational engagement with Asian residents in long-term care facilities: a mixed method systematic review

**DOI:** 10.3389/fpubh.2024.1422134

**Published:** 2024-07-16

**Authors:** Hao Liu, Anne Topping, Ping Guo

**Affiliations:** ^1^University of Birmingham, School of Nursing and Midwifery, Institute of Clinical Sciences, College of Medical and Dental Sciences, Birmingham, United Kingdom; ^2^University Hospitals Birmingham NHS Foundation Trust, Birmingham, United Kingdom

**Keywords:** intergenerational engagement, older people, loneliness, social isolation, quality of life, mixed method, systematic review, patient and public involvement

## Abstract

**Introduction:**

Asian countries are experiencing a rapid rise in their aging populations. Cognitive and physical decline associated with aging can limit social interaction. This particularly impacts on those residing in long-term care facilities and engagement with children and young people. Intergenerational engagement has known benefits on the health and wellbeing of older people, it is unclear what the impact of intergeneration engagement interventions might have on older people in Asian long-term care settings. This review aims to evaluate the effectiveness and experiences of intergenerational engagement with older people in long-term care facilities in Asia.

**Methods:**

Ten databases were searched to locate empirical studies of any design published in English or Chinese from January 2000 to June 2023. The search was limited to papers reporting effectiveness and/or experiences of intergenerational engagement on older people residing in Asian long-term care settings. The protocol was registered with PROSPERO (CRD42023413935) and followed PRISMA guidelines for reporting. A convergent design employing narrative synthesis was used to synthesize and integrate findings.

**Results:**

From initial searches, 1,092 records were identified, of which 13 studies were retained for the review: 7 quantitative (including 1 randomized controlled trial, 1 cross-sectional observational design, and 5 quasi-experimental designs), 3 qualitative, and 3 mixed methods. Included studies were of variable quality. Quantitative evidence revealed that intergenerational engagement reduced depression (4.47 vs. 8.67, *p* = 0.005), negative emotions (14.11 vs. 16.56, *p* = 0.030), and feelings of loneliness (*p* < 0.01) among older people; and increased quality of life (mean change = −1.91; 95% CI = −3.18, −0.64) and strengthens interpersonal interactions (*p* = 0.025). Qualitative insights suggested that intergenerational engagement could foster emotional bonds, enhance intergenerational relationships, promote lifelong learning, satisfy social needs and improve older peoples’ overall quality of life. However, some challenges such as language differences and noise levels can hinder successful implementation of intergenerational engagement.

**Conclusion:**

This review indicates that intergenerational engagement can reduce depression and loneliness, improve quality of life, and strengthen social bonds for older individuals in Asian long-term care facilities. Despite some challenges, the evidence underlines its potential to meet the emotional and social needs of older people. Recognizing and addressing delivery challenges is essential for effective implementation.

**Systematic review registration:**

https://www.crd.york.ac.uk/prospero/display_record.php?ID=CRD42023413935, identifier: CRD42023413935.

## 1 Introduction

Asia is witnessing a significant demographic shift with an increasing aging population. The percentage of the population aged 65 or older in Eastern and Southeast Asia is estimated to increase from approximately 13% in 2022 to 26% by 2050, effectively doubling in the next 18–26 years (1). This presents significant challenges to policy makers and health and social care planners not least time to prepare, allocation of resources, and workforce availability and expertise to care for older people compared to Western nations (2). Concomitant with this rapid increase in an aging population is the increasing demand for long-term care, and burden on health and well-being services across Asia. In response, several Asian countries such as China, South Korea, Singapore and Japan and cities have developed long-term care systems to accommodate the care and support needs of older people including increasing provision of nursing homes, residential and sheltered housing and daycare centers (3–5).

Aging is a complex process affecting people differently and not necessarily chronologically While older people may possess wisdom and enhanced decision-making abilities acquired experientially, they can also face physical and cognitive decline, susceptibility to mental ill-health, and social isolation (6–10). Evidence suggests that social interaction plays a critical role in mitigating some of the negative aspects of aging (11, 12). Older people can benefit from enhanced social and intellectual engagement (13), and robust social networks, and consistent social engagement are pivotal for well-being (14, 15).

Intergenerational Engagement (IE) is defined as ‘an organized initiative that brings together people from different age groups, typically older people and children and young people (CYP), to provide benefits to all participants involved’ (16). Previous studies particularly from North America and other Western countries have shown a variety of potential benefits of IE in improving older people’s physical and psychological health, socialization, sense of self-worth, and independence (17–20). Various IE interventions have been tested as an approach for increasing social interaction and demonstrated some benefits (21–26). Likewise previous reviews have underscored some e potential benefits of IE among older people (19, 27). There remains a lack of evidence of the potential benefit of IE for older people living in Asian long-term care facilities.

Asia is renowned for its vast geographical and cultural diversity, spanning numerous countries and landscapes (28). In the context of this review, Asia is defined as a region encompassing Eastern Asia, Southern Asia, and Southeastern Asia. These areas, characterized by shared cultural foundations shaping societal perspectives on aging, older adult care, and family structure (29, 30), are experiencing rapid aging populations, requiring innovative long-term care solutions (31). By focusing on these areas (Eastern Asia, Southern Asia, and Southeastern Asia), IE in long-term care facilities can enhance the health and well-being of older people, informed by similar cultural, demographic, and social contexts.

## 2 Aim and objectives

This review aimed to evaluate the effectiveness and experiences of IE with older people in long-term care facilities in Asia. The specific objectives were:

- To evaluate the effectiveness of IE on older people in long-term care facilities in Asia.
- To identify the health outcomes and measurement tools used to assess the effectiveness of IE among older people living in long-term care facilities in Asia.
- To analyze the key components of various IE interventions used in studies conducted with older people residing in long-term care facilities in Asia, including CYP’s age groups, activity designs, durations, and frequency of contact.
- To explore the experiences of older people participating in IE in Asian long-term care facilities.

## 3 Methods

This systematic review used a convergent synthesis design (32). We adopted the Preferred Reporting Items for Systematic Reviews and Meta-Analyses (PRISMA) guidelines (33) and registered the protocol with PROSPERO (CRD42023413935).

### 3.1 Search strategy

A comprehensive literature search was conducted between April and June 2023. Ten electronic databases were searched, including PubMed, MEDLINE, Web of Science, CINAHL, PsycINFO, Embase, Cochrane Central Register of Controlled Trials (CENTRAL), China National Knowledge Infrastructure (CNKI), Wan Fang Database, and Airiti Library, complemented by additional searches in Google Scholar (34). Reference lists from relevant studies were also manually searched. The search strategy focused on three principal keyword categories: ‘intergenerational engagement’, ‘older people’, and ‘long-term care facilities’. To incorporate Chinese literature, both Simplified and Traditional Chinese search terms were utilized. The search strategy involved using the Boolean operator “OR” to combine keywords within each category, and “AND” to link the categories (35). The keyword categories and search terms are presented in Table 1. The term ‘Asia’ was not used as a keyword to prevent excluding relevant studies that did not specify the location in their titles or abstracts. Studies conducted outside Asia were excluded, those lacking specific location indicators underwent a full-text review to determine their relevance to the Asian context. Before beginning the searches, the strategies were reviewed and checked against the Peer Review of Electronic Search Strategies (PRESS) Guidelines with a librarian to assure the approach (36).

**Table 1:** The keyword categories and search terms in English, simplified Chinese, and traditional Chinese.

Keyword categories	Search terms (English)	Search terms (Simplified Chinese)	Search terms (Traditional Chinese)
Intergenerational engagement	“Intergenerational Relations”[MeSH] or ((intergeneration* or inter-generation* or crossgeneration* or cross-generation* or multigeneration* or multi-generation*) adj3 (engage* or program* or interact* or learn* or care* or caring or activit* or practice* or exchang*))	代际互动 or 代际交流 or 代际合作 or 代际活动 or 代间学习 or 代间互动 or 跨代沟通 or 跨代交流 or 跨代合作 or 跨代学习 or 跨代互动 or 跨代支持	代際互動 or 代際交流 or 代際合作 or 代際活動 or 代間學習 or 代間互動 or 跨代溝通 or 跨代交流 or 跨代合作 or 跨代學習 or 跨代互動 or 跨代支持
Older people	“Aged”[MeSH] or “Retirement”[MeSH] or older people or older* or elder* or senior* or geriatric* or retirement or old* adult* or aging or aging or old* people or old* person*	老年人 or 老人家 or 长者 or 老年居民 or 老年群体 or 老年个体 or 退休人员 or 年迈者 or 高龄者	老年人 or 老人家 or 長者 or 老年居民 or 老年群體 or 老年個體 or 退休人員 or 年邁者 or 高齡者
Long-term care facilities	“Long-term care”[MeSH] or “Nursing Homes”[MeSH] or “Homes for the Aged “[MeSH] or long-term care or nursing home* or residential care or assisted living or care home* or homes for the aged or skilled nursing facilit* or continuing care retirement communit* or older adult care facilit* or long-term care institution* or geriatric care center* or senior living communit* or retirement home* or aged care facilit* or convalescent home* or rest home* or old people’s home* or elder care center* or geriatric residential facilit*	养老院 or长期护理 or 长期照护 or 护理院 or 敬老院 or安养院 or疗养院or退休院or养老机构 or 养老社区 or 老年人照顾 or 老年护理中心 or 老年社区 or 老年居住社区 or 老年福利院 or 老年之家 or 医养结合中心 or 长者之家or 老年剬寓	養老院 or長期護理 or 長期照護 or 護理院 or 敬老院 or安養院or療養院or退休院or養老機構 or 養老社區 or 老年人照顧 or 老年護理中心 or 老年社區 or 老年居住社區 or 老年福利院 or 老年之家 or 醫養結合中心 or 長者之家or老年剬寓

### 3.2 Eligibility criteria

#### 3.2.1 Inclusion criteria

Studies were included if they met the following criteria:

1. Participants were older people aged 60 or above, with or without dementia, residing in Asian long-term care facilities (e.g., such as nursing homes, care homes, retirement homes, geriatric facilities, and daycare centers). Despite the global standard for classifying older people as 65 years and above, our study defined older people as aged 60 and above, adjusting for the varied definitions in some Asian countries (37).
2. Studies that focused on IE involving older people and children or young people and examined the effectiveness and/or experiences of IE with older people.
3. Empirical studies, including quantitative, qualitative, and mixed method designs, published in English or Chinese from 2000 onwards.

#### 3.2.2 Exclusion criteria

The following studies were excluded from the review:

1. Studies that did not involve IE between older people and children or young people.
2. Studies that only evaluated the effectiveness and/or experiences of IE of children and/or young people, caregivers, or staff.
3. Studies conducted outside of long-term care facilities (such as schools, and hospitals), and/or outside of Asia.
4. Secondary research (such as secondary analysis and reviews), editorials, expert opinions, and conference proceedings.
5. Studies published in languages other than English or Chinese.

### 3.3 Study selection

Search results were imported into EndNote 20, following the removal of duplicates, the remaining references were transferred to Rayyan (38) for further screening. Titles and abstracts were screened to exclude irrelevant studies, and the full texts of the remaining studies were retrieved and assessed against the inclusion and exclusion criteria. The first reviewer (HL) scrutinized all the records according to preset inclusion and exclusion criteria. The other two reviewers (AT & PG) independently scrutinized half of the records each. During the stages of the selection process, any conflict or disagreement between the two reviewers (HL & PG) was solved through discussion or consultation of a senior reviewer (AT).

### 3.4 Quality appraisal

The risk of bias in the studies was evaluated using the Mixed Methods Appraisal Tool (MMAT) (39). Each study, whether qualitative, quantitative, or mixed methods, was evaluated according to specific criteria - five items for qualitative or quantitative studies and 15 items for mixed methods studies. Each included study was appraised by HL and subsequently double-checked by AT and PG. Quality scores were not calculated in line with the approach recommended by the developers of MMAT (39). No studies were removed based on quality assessment due to their potential of all included studies to contribute insights.

### 3.5 Data extraction and synthesis

HL designed the data extraction table, performed the data extraction, and AT and PG checked extraction. The extracted data included the author, year of publication, study design, location, sample size, characterization of participants, design of the intervention (e.g., age groups of the younger generation, type of activity, durations, and frequency), and findings. A convergent synthesis approach was applied to analyze both quantitative and qualitative evidence, then integrated to evaluate the effectiveness of IE on health and well-being (32). Quantitative data were synthesized narratively and presented in tables, while qualitative insights were distilled through meta-aggregation, emphasizing participant experiences. Meta-aggregation was chosen for its structured and rigorous approach for the integration of qualitative data; ensuring a comprehensive and accurate synthesis for well-founded recommendations for practice and research (40). After extraction, study results were grouped by conceptual similarities, merged into key concepts, and integrated into overarching synthesized findings expressed as themes (41).

## 4 Results

### 4.1 Studies identified

**Figure 1:**
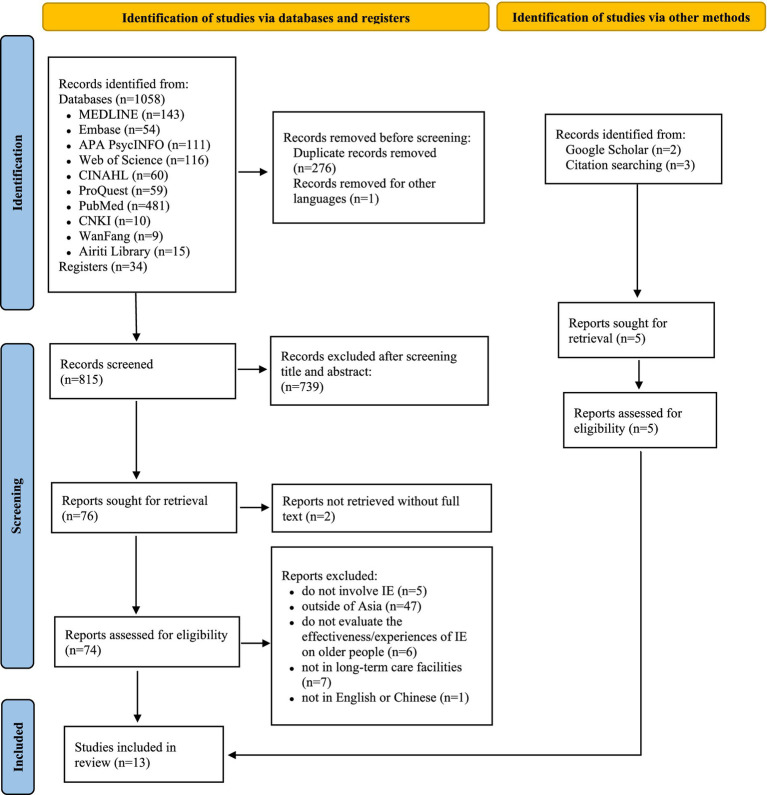
PRISMA flow diagram depicting the study selection process at each review stage.

A total of 1,092 studies were initially identified from databases (*n* = 1,058) and registers (*n* = 34). After removing duplicates (*n* = 276) and records in other languages (*n* = 1), 815 studies were screened by title and abstract and 739 were excluded. Originally 76 were identified for full-text retrieval, though two could not be sourced. Of the remaining 74 full-text studies assessed, 66 were excluded for the following reasons: did not involve IE (*n* = 5), outside of Asia (*n* = 47), did not evaluate the effectiveness or experience of IE on older people (*n* = 6), outside long-term care facilities (*n* = 7) and the full text was not available in English or Chinese (*n* = 1). Five additional studies were found through other methods: two studies were identified through Google Scholar, and three studies by performing citation searching. Finally, 13 studies were included in the review (42–54). The PRISMA flow diagram depicting the study selection process at each review stage is shown in Figure 1.

### 4.2 Studies quality appraisal

The assessment of the 13 studies using the MMAT criteria showed variations in the quality of the studies, with the average rating being moderate. Some studies demonstrated robust methodological rigor (43, 45, 48, 52–54), and a few showed significant areas for improvement, especially in meeting the mixed methods criteria. MMAT assessments for all included studies are shown in Supplementary Table 1.

### 4.3 Characteristics of included studies

The studies employed a range of research methods: seven quantitative studies (one randomized controlled trial (44), one cross-sectional observational study (50), and five quasi-experimental designs (42, 43, 47–49)), three qualitative studies (52–54), and three studies using mixed methods (42, 46, 51). These 13 studies were conducted in Mainland China (*n* = 4), Taiwan (*n* = 5), South Korea (*n* = 1), Hong Kong (*n* = 1), Singapore (*n* = 1), and Japan (*n* = 1). Among the included studies for review, six studies were written in English and seven studies in Chinese. The characteristics of the included studies are summarized in Table 2.

**Table 2:** Characteristics of the included studies.

Study	Location	Design	Participants (Older people)	Participants (CYP)	Intervention (Activity)	Duration and frequency	Key findings
Wang (2023) (44)	Mainland China	RCT	*n* = 38 EG:20 CG:18 Aged: 58–91	Nursery children *n* = 40 Aged: 4–5	EG: one-to-one social interactions (e.g., educational activities, games, and art) + watch children’s performances; CG: watch children’s performances	6 weeks; 5 days a week (20 min)	One-to-one social interactions between older people and young children enhanced older people’s physical health and psychological well-being in a Chinese context.
*Wang and Wang (2022) (45)	Mainland China	Mixed method	*n* = 37 EG:19 CG:18 Aged: 70 ~ 98	Nursery children *n* = 20 Aged: 4–5	Based on the interests of the older adult and children (e.g., pickled vegetables)	6 weeks; 5 days a week (20 min)	IE between older people and young children significantly enhanced older people’s positive beliefs and well-being while reducing negative emotions. Close relationships were the way IE positively affected their mental health.
*Li et al., (2022) (42)	Mainland China	Quasi-experimental design	*n* = 57 IG:28 M_aged_ = 76.89 ± 4.95 CG: 29 M_aged_ = 75.38 ± 4.98	Preschool children Aged: NA	Stimulating interactions, aging learning sessions, sociocultural activities (e.g., singing, playing games)	2 months once a week (120 min)	IE effectively reduced depression and loneliness among older people in the long-term care facility and increased their subjective well-being; however, no improvement in their quality of life was observed.
*Tsai and Lin (2022) (48)	Taiwan	Quasi-experimental design	*n* = 28 Aged≥65	Nursery children *n* = 29 Aged: 4–6	7 themed intergenerational learning (assisted by Zenbo intelligent robots)	6 weeks; once a week (60 min)	Intergenerational learning effectively alleviated depression in older people, but it did not significantly improve their cognitive function.
Leong et al. (2021) (52)	Singapore	Qualitative	*n* = 18 Aged:68–94	students *n* = 20 Aged: 13–16	Semi-structured, student-led creative and social activities or performances	4 weeks; once a week; (90 min)	IE fostered social interactions, companionship, and mutual care between older people and young people, promoting their active engagement and development.
*Wang (2020) (54)	Mainland China	Qualitative	*n* = 500 M_aged_ = 84	Youth volunteers *n* = 30 Aged: NA	Living together (youth volunteers provide educational/ companionship services; group/ thematic activities)	2 years; (no less than 20 h per month)	Engaging older people in the IE program enhanced their technological skills, adapted them to modern life, enriched their cultural and spiritual well-being, and improved their quality of life through intergenerational support and cultural exchange.
Kim and Lee (2018) (43)	South Korea	Quasi-experimental design	*n* = 60 EG: 30 CG:30 Aged: most≥80	Second-year high school students *n* = 60 Aged: NA	30 min of positive interactions (e.g., making picture frames, singing, and painting); 40 min of reminiscence therapy; 20 min of reflection	6 weeks once a week (90 min)	The IE program helped older people rediscover the value of their lives, develop positive feelings about their lives, and adapt to circumstances through sustained positive interactions with the younger generation.
*Hong and Yao (2017) (47)	Taiwan	Quasi-experimental design	*n* = 10 Aged≥60	Nursery children *n* = 10 Aged: 6	5 themed intergenerational learning	5 weeks; once a week (60 min)	Intergenerational learning improved older people’s interpersonal interactions, helped them connect better with young children, and enabled them to learn from different generations.
Hwang et al. (2014) (51)	Taiwan	Mixed method	*n* = 66 EG:33 CG:33 Aged≥60	Nursing students *n* = 250 M_aged_ = 18 (2 students with 1 older people)	Intergenerational service-learning: life story sharing through artwork; recreational activities	10 weeks; once a week (120 min)	With sufficient training and clear expectations, a well-designed IE program met older people’s needs for care and social contact by improving intergenerational interactions.
Morita and Kobayashi (2013) (50)	Japan	Quantitative cross-sectional observation design	*n* = 25 performance-base:11; social-oriented:14 Aged:71–101	Preschool children *n* = 60 Aged: 5–6	①performance-based: watching children’s performances; ②social-oriented: play games together	3program, each program last 1 month; once or twice a month (20–30 min)	IE with preschool children brought smiles and conversation to older people, allowing them to play more roles and fulfill social needs, thereby reintegrating them into society.
*Fan (2010) (53)	Taiwan	Qualitative	*n* = 6 Aged: NA	Young collaborators *n* = 6–7 Aged:26–30	11 themed art workshops +1 review session	12 weeks; once a week (90 min)	The IE art program fostered positive intergenerational relationships, enhanced older people’s motivation to participate in activities, and enriched their lives in long-term care facilities.
Chung (2009) (49)	Hongkong	Quasi-experimental design	*n* = 49 M_aged_ = 79 ± 6.05	Young people *n* = 117 Aged: 16–25 (2–3 young people with 1 older people)	Older people share and discuss life experiences, with youth aiding in the creation of personalized life storybooks	12 weeks; once a week (90 min)	The intergenerational reminiscence program had a positive impact on the quality of life of older people with dementia and reduced their depression levels.
*LinOu (2004) (46)	Taiwan	Mixed method	*n* = 24 (all female) EG:12 CG:12 M_aged_ = 77.4	Nursery children *n* = 12 Aged: 5–6	12 themed art workshops	6 weeks; twice a week (30–40 min)	Although there was no difference in intergenerational satisfaction, the interaction and cooperation between older people and children became more harmonious and mutually understanding with the increased frequency of IE activities.

EG, Experimental group; CG, Control group. *Studies written in Chinese.

The studies featured sample sizes ranging from six to five hundred older people aged between 60 and 101. While several studies included older people who were able to communicate and were physically healthy, one study also included older people with dementia (49), and two other studies involved older people with cognitive impairments (50, 53).

### 4.4 Design of intergenerational engagement

#### 4.4.1 Intergenerational engagement structure

The duration of the IE programs in the reviewed studies varied from 4 weeks to 2 years, with 6 weeks being the most common duration (43–46, 48). In one study, a 10-week IE program was structured as follows: 3 weeks of student training, 6 weeks of interaction between older people and students, and 1 week for presentations (51). Most studies favored weekly sessions (42, 43, 47–49, 51–53), two studies opted for daily interactions (44, 45), one study conducted sessions twice a week (46), and another once per month (50). The length of each session ranged from 60 to 90 min, with a few studies incorporating shorter (20–30 min) (44, 45) or longer sessions (120 min) (42, 51). One study instead of fixed session times, young volunteers and older individuals engaged in IE for a flexible 20 h each month, without adhering to a predetermined schedule (54). However, no studies examined how the delivery structure, including the duration and length, of IE programs affected outcomes.

#### 4.4.2 Types of activities

A wide range of IE activities were described but all were designed to enhance the wellbeing of older people in Asian long-term care facilities. These ranged from interactive social and cultural activities, such as singing and games, to more structured programs involving reminiscence therapy and artistic expression through themed events. One study incorporated novel technology by using intelligent robots for sensory and memory games (48); another focused on creating supportive environments for older people with dementia to share life experiences (49). Combinations of educational and recreational activities were also common, often facilitated by young people in service-learning capacities, including life story sharing, performance arts, and thematic workshops (43, 51, 54). These programs collectively highlighted the variation of IE initiatives designed to address the mental, emotional, social, and cognitive needs of older people. Among the 13 studies reviewed, no studies employed patient and public involvement (PPI) in developing the IE programs or design of the research studies.

#### 4.4.3 The age groups represented in studies

The age range of the CYP in the 13 studies reviewed spanned from early childhood to young adulthood. Early childhood, encompassing nursery and preschool children typically aged 4–6 years in the Asian region, was the focus of seven studies (42, 44–48, 50). One study examined a group of students aged 13–16 years (52), and another included second-year high school students without specifying their ages (43). Although the students’ ages were not specified, high school students are typically 16–18 years old in South Korea where the study was conducted (55). Additionally, four studies extended the age spectrum: one included young participants aged between 16 and 25 years (49), another focusing on nursing students aged 18 (51), one study involved young collaborators aged 26–30 years (53), and the final study included youth volunteers without specifying their ages (54). Although IE programs may offer benefits to both younger and older age groups (16), our review specifically focused on evaluating the effectiveness and experiences of these programs among older people. This approach is in strict alignment with the aims and objectives outlined in our review protocol.

#### 4.4.4 Pre-training

Researchers in two studies (43, 51) reported that the children and young people (CYP) participants received pre-training before engaging with older people, and only one study (42) mentioned that the long-term care facility staff received pre-training before the program. Kim and Lee (43) described providing two pre-training sessions for the CYP that included information about aging, characteristics of older people, and communication skills, with each session lasting 50 min. Another study (51) described pre-service training consisting of a six-hour session emphasizing self-introduction and life story sharing activities, communication skills, and developing empathy toward older people. The staff pre-training included understanding the IE program, its theoretical basis, implementation skills, characteristics of older people, and emergency protocol (42). However, these studies did not examine the impact of pre-training on implementing the IE program and the outcomes.

### 4.5 Quantitative evidence

#### 4.5.1 Measurement tools

To evaluate the effectiveness of interventions, researchers employed a comprehensive array of measurement scales. Physical health was assessed through both self-rated and interviewer-rated health assessments, along with evaluations of disability in daily living activities. Mental health assessments utilized various versions of the Geriatric Depression Scale, the Short-form UCLA Loneliness Scale, and the Center for Epidemiological Studies Short Depression Scale, among others, to gage aspects of mental well-being. The Mini-Mental State Examination (MMSE) was used to measure cognitive function, while social interaction was evaluated through the Intergenerational Satisfaction and Interpersonal Interaction Function Scales. Quality of life was assessed using the Quality of Life in Alzheimer Disease Scale, and adaptability to long-term care settings was measured with the Nursing Home Adaptation Scale and the Older adult Resident-Perceived Caring Scale.

A significant focus of the studies was on mental and emotional well-being, which was examined using scales that measured depression (42, 44, 45, 48, 49, 51), loneliness (42), happiness (42, 44, 45), and emotions (43–45). Additionally, some studies also looked into social interaction (46, 47), quality of life (42, 49), adaptability (43, 51), cognitive function (48, 49), and physical health (44). The diverse scales employed in these studies provided a nuanced understanding of the IE’s effectiveness. The specific measurement tools and the results on the effectiveness of IE are presented in Table 3.

**Table 3:** The measurement tools and the results on the effectiveness of IE.

Study	Measurement tools						Results*
	Physical	Mental	Cognitive	Interaction	Quality of life	Adaptability	
**Li et al., (2022) (42)		①Chinese version of the Geriatric Depression Scale (30-item); ②Short-form UCLA Loneliness Scale; ③Memorial University of Newfoundland Scale of Happiness			Quality of Life in Alzheimer Disease		Post-test, EG vs. CG depression:12.90 vs. 15.41 (*p* < 0.01); loneliness: 12.59 vs. 16.50 (*p* < 0.01); subjective wellbeing 26.8 vs. 23.18 (*p* < 0.01); quality of life: no significant differences (*p* > 0.05).
Kim and Lee (2018) (43)		①Korean version of the Positive Affect Negative Affect Schedule; ② Korean version of the ego integrity scale				Korean version of the Nursing Home Adaptation Scale	After the program, the EG scored: ego integrity improved from 100.63 to 112.93 (*p* < 0.001); positive emotion increased from 49.27 to 54.80 (*p* = 0.001); nursing home adaptation moved from 74.43 to 83.73 (*p* < 0.001).
Wang (2023) (44)	①Self-rated physical health; ②Interviewer-rated health; ③Disability in activities of daily living	①Center for Epidemiological Studies Short Depression Scale; ②Rosenberg Self-Esteem Scale; ③General Self-Efficacy Scale; ④Negative affect; ⑤Flourishing Scale					After the intervention, the EG showed: a decline in self-rated health by −1.000 (*p* = 0.011) and depression by −2.368 (*p* = 0.042); improvements in self-efficacy by 2.316 (*p* < 0.001) and flourishing by 6.526 (*p* = 0.026); Disability in activities of daily living, interviewer-rated health, and self-esteem had minor or no significant changes.
**Wang and Wang (2022) (45)		①Center for Epidemiological Studies Short Depression Scale; ②Rosenberg Self-Esteem Scale; ③General Self-Efficacy Scale; ④Negative affect; ⑤Flourishing Scale; ⑥Peace of Mind Scale					Post-test, EG vs. CG self-efficacy: 29.68 vs. 22.61 (*p* < 0.001); self-esteem: 30.11 vs. 27.83 (*p* = 0.062); peace of mind: 30.74 vs. 26.28 (*p* = 0.001); flourishing: 48.42 vs. 38.22(*p* < 0.001); depression: 4.47 vs. 8.67 (*p* = 0.005); negative emotion: 14.11 vs. 16.56 (*p* = 0.030)
**LinOu (2004) (46)				Intergenerational Satisfaction Scale			Intergenerational satisfaction scores: no significant difference between EG and CG (F = 0.68, *p* > 0.05).
**Hong and Yao (2017) (47)				Interpersonal Interaction Function Scale			Interpersonal interaction function improved from 13.4 to 10.33. significant improvements were observed in “I do not like to approach crowds” (pre-test: 2.11, post-test: 1.33, *p* = 0.023) and “I always prefer to go to places where there are no people when I’m free” (pre-test: 2.00, post-test: 1.33, *p* = 0.004).
**Tsai and Lin (2022) (48)		Chinese version of the Geriatric Depression Scale (15-item)	Chinese version of Mini-Mental State Examination				After the intervention, depression: decreased from 17.27 to 10.79 (*p* = 0.0007); cognitive function: no significant differences (*p* = 0.3451).
Chung (2009) (49)		Chinese version of the Geriatric Depression Scale (15-item)	Chinese version of Mini-Mental State Examination		Quality of Life in Alzheimer Disease		After the intervention, quality of life: scores increased from 32.12 to 35.41 (mean change = −1.91; 95% CI = −3.18, −0.64); depression: scores decreased from 8.10 to 6.88 (mean change = 1.86; 95% CI = 0.92, 2.80); cognitive function: no significant differences.
Morita and Kobayashi (2013) (50)				Observation record form***			The social-oriented program excelled in constructive behavior and conversation compared to the performance-based program(*p* < 0.001); Only the weighted smiling rate was higher in the social-oriented program (*p* < 0.05); The performance-based program had superior visual attention between generations (*p* < 0.05).
Hwang et al. (2014) (51)		Well-being Picture Scale				Older adult Resident-Perceived Caring Scale	Post-test, EG vs. CG Older adult Resident-Perceived Caring Scale: 60.31 vs. 53.01 (*p* < 0.01); wellness: no significant differences.

IE, Intergenerational Engagement; EG, Experimental group; CG, Control group. *IE programs may benefit both young and old, but our review only extracted data related to older people. **Studies written in Chinese. ***Observation record form including visual attention; facial expression; engagement/behavior; intergenerational; conversation.

#### 4.5.2 Effectiveness of intergenerational engagement

##### 4.5.2.1 Mental and emotional well-being

The interventions demonstrated a significant reduction in depression scores across various studies (4.47 vs. 8.67, *p* = 0.005; 12.90 vs. 15.41, *p* < 0.01; 10.79 vs. 17.27, *p* = 0.0007), demonstrating the effectiveness of the IE programs in alleviating depressive symptoms (42, 45, 48). A significant reduction in loneliness was observed (12.59 vs. 16.50, *p* < 0.01) in one study (42). IE was also associated with a decrease in negative emotions (14.11 vs. 16.56, *p* = 0.030) (45) and an increase in positive emotions (49.27 to 54.80, *p* = 0.001) (43). These outcomes, coupled with increased self-efficacy, peace of mind, flourishing, and subjective well-being (44, 45), underscore the comprehensive benefits of IE on mental and emotional well-being.

##### 4.5.2.2 Social interaction

IE led to a significant improvement in interpersonal interaction functions, as evidenced by a reduction in scores from 13.4 to 10.33 (*p* = 0.025) (47). In this context, a lower score not only indicated better outcomes but was also accompanied by a marked increase in social comfort. Another study (50) found that social-oriented programs (play games together) rather than performance-based (watching children’s performances) enhanced constructive behavior and conversation (*p* < 0.001) and increased smiling frequency (*p* < 0.05). In contrast, performance-based programs improved visual attention (looked at each other) between two generations (*p* < 0.05) (50). However, one study (46) found that there was no significant difference in intergenerational satisfaction between the experimental and control groups (*F* = 0.68, *p* > 0.05).

##### 4.5.2.3 Quality of life and adaptability

A study with one group design (49) observed a significant increase in quality of life scores, indicating a difference before and after the intervention, rising from 32.12 to 35.41 (mean change = −1.91; 95% CI = −3.18, −0.64). However, another study (42) reported no significant change in quality of life between the intervention and control groups after the intervention (*p* > 0.05). These divergent outcomes may stem from variations in intervention design, including duration and the age of the young participants, which could have further contributed to the observed disparities in outcomes between the studies. Furthermore, differences in study design, such as employing a one-group design versus comparing intervention and control groups, may influence outcomes.

IE enhanced the adaptability to institutional life and perceived care quality for older people in long-term care facilities, with the adaptation score increasing from 74.43 to 83.73 (*p* < 0.001) (43). There was a difference in the scores on the older adult resident-perceived caring scale, developed to measure the general caring behaviors perceived by residents of long-term care facilities, between the experimental and control groups after IE (60.31 vs. 53.01, *p* < 0.01) (51).

##### 4.5.2.4 Cognitive function and physical health

In relation to cognitive functioning among older people, one study demonstrated that MMSE scores increased slightly from 17.17 to 17.89 (*p* = 0.3451) but no statistical significance was found (48), and another study also reported no significant differences (49). Among 13 studies, only one focused on physical health, noting a decline in self-rated health (by −1.000, *p* = 0.011) with little to no change in activities of daily living and interviewer-rated health (44).

### 4.6 Qualitative evidence

**Figure 2:**
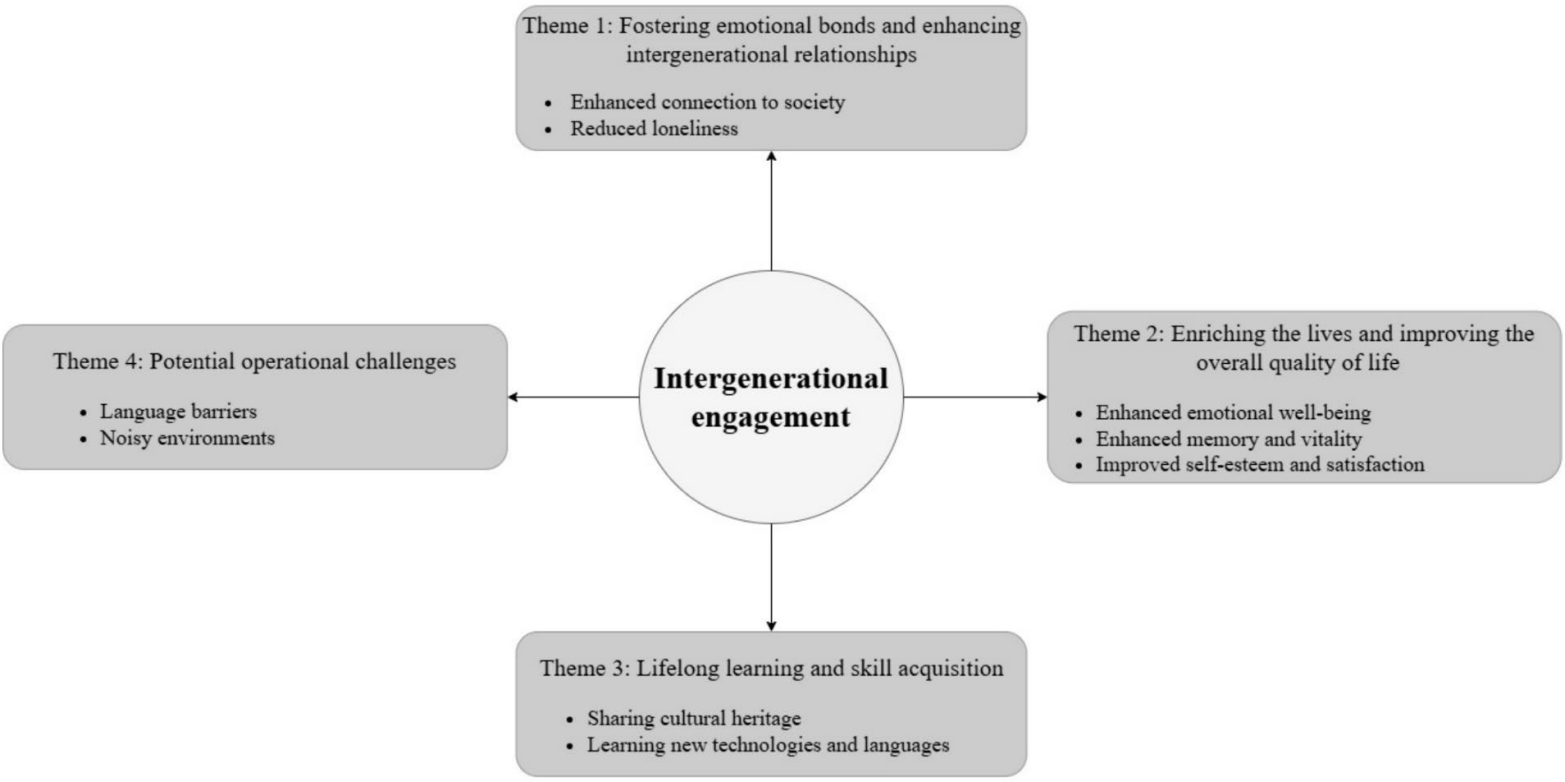
Themes identified from qualitative evidence.

The qualitative findings of this review are organized into four key themes, as illustrated in Figure 2.

#### 4.6.1 Theme 1: Fostering emotional bonds and enhancing intergenerational relationships

IE appeared to foster emotional support and the opportunity for meaningful relationship engagement among older people, countering the isolation that can accompany aging and/or life in institutional settings. Sustained engagement in interactive activities seemed necessary for establishing and maintaining these relationships. *“As the number of activities and interactions increases, the intergenerational relationships between the older adult and children mostly develop towards reciprocity and positivity* (46)*.”* Participants (older people) perceived IE positively, as enhancing their emotional well-being and fostering connections with the CYP, thereby bridging generational gaps. *“Because it makes me happier when I am with them. I am a little more positive. Just stronger emotionally* (52)*.”* Establishing relationships and emotional bonds with the CYP appeared to be central to any benefits gained from IE for the older people. IE appeared to both counter feelings of loneliness and enrich the environment within care facilities. *“Often in the mornings, we see young people jogging, instantly filling the nursing home with energy. Sometimes we also meet them in the dining hall, and they join us for meals. This daily companionship is precious and quite wonderful* (54)*.”* The physical presence and interaction with CYP may underscore the potential role that IE could play in fostering connections between different generations.

#### 4.6.2 Theme 2: Enriching the lives and improving the overall quality of life

IE programs appear to play a significant role in enriching the lives of older people and improving their overall quality of life. Intergenerational engagement creates a space for interactive activities and shared experiences that enrich the lives of older people within long-term care facilities. *“I like people to come and live(n) things up. If you look around, you can see that people here do not talk with each other a lot* (51)*.” “Because they bring joy to us* (52)*.” “Young volunteers living in here have brought us much joy, seeing them is like seeing sunshine* (54)*.”* Participation in IE programs involving social and physical activities can also lead to beneficial outcomes for older people, including improved self-esteem (45) and satisfaction (52), increased vitality (54), and enhanced memory (52). *“Students let us guess things from the screen (memory games), we have to memorize the items very fast. I can still remember the next day; our brains are very good* (52)*.”* Interacting with younger generations can lessen negative feelings about aging, helping older people feel younger. *“I hope students come often because they show us how to draw and they hold fun activities that make us laugh. They make me feel young again and forget the aged life…* (51)*.”* IE seemed to reduce feelings of loneliness in the older people and enhance their sense of connection to the community outside of long-term care settings. *“After talking to them I feel like I have directly integrated into this society* (52)*.”* From the accounts it would seem IE brings an externality that enables connection with what is happening beyond the confines of the institution. Evidence from these studies (51, 52, 54) highlights the impact of IE programs, which not only may reduce loneliness among older people in long-term care facilities but also enhance a sense of connection to society, thereby feeling more integrated and enriched.

#### 4.6.3 Theme 3: Lifelong learning and skill acquisition

IE provided a platform for reciprocal learning and skill development. Through engagement with CYP participants, older people were able to learn how to use technologies, such as smartphones and computers, thereby increasing their engagement with modern life. *“Now that we have learned to use smartphones, it’s not just young people who can surf the internet; even us in our eighties have picked it up, feeling like we can keep up a bit with the younger generation’s pace, and instead of feeling clumsy as before, we now feel smarter* (54).*”* Engaging in traditional arts like handicrafts allows both CYP and older people to connect through sharing their cultural heritage and learning new languages, such as English, including common expressions. *“The children do handicrafts with us, make the bird, boat. Use paper to do* (52)*.” “I used to only hear a few words of English on TV, but now, with young volunteers teaching us, I can recognize some letters and even speak a few words of English – it feels great* (54)*.”* Through the acquisition of new skills, it seemed older people gained a sense of self-worth, identity, and joy, which collectively enhanced their overall quality of life and well-being. Participating in art workshops allowed older people to revisit hobbies and skills, providing opportunities to rekindle past interests and share these experiences with the younger generation. *“I had not painted since graduating from primary school, until this activity…* (53)*.”* These findings suggest that IE programs provide an opportunity for lifelong learning, bridging the generational gap through the mutual exchange of skills and experiences. Such initiatives and reciprocity promoted learning as a continuous process that appeared to be affirming, contributing to quality of irrespective of age.

#### 4.6.4 Theme 4: Potential operational challenges

While IE offers potential benefits for older people, there are still some operational challenges such as language differences and noisy environments that require attention for these programs to be successfully implemented. Language differences can hinder the development of meaningful social connections, as communication is the foundation of social engagement and mutual understanding. *“(Communication is) very difficult (with students). They do not speak Chinese. They do not understand the language I am saying* (52)*.”* Similarly, noisy environments may be challenging for older people, particularly if experiencing hearing loss, and impede the quality of interaction between different generations. *“I disliked students gathering in noisy groups. And talking nonsense. They should speak softly or chat with the residents outside instead of making so much noise talking and laughing. This is a place for rest* (51)*.”* These findings underscore the need for IE programs to be well-planned and located in appropriate accommodations in long-term care facilities so a broad range of activities can be delivered in a context that does not contribute to isolation and meets the needs and capabilities of older people.

## 5 Discussion

This systematic review identified 13 studies that reported the effectiveness and experiences of IE among older people in long-term care facilities in Asia. These studies revealed that IE can enhance the mental and emotional well-being, social interaction, and quality of life of older people. Notably, IE was associated with substantial reductions in depression (44, 45) and loneliness (42), and increase in positive emotions (43) and self-efficacy (45). When planned considerately and executed thoughtfully, these programs can support dynamic relationship building, enabling older people to overcome any isolation associated with aging and foster a positive environment within long-term care facilities. The studies also emphasized the value of lifelong learning, highlighting how older people can benefit from acquiring new technological skills and engaging in cultural activities, that contribute to a richer sense of identity and enjoyment.

The review has also uncovered potential challenges to effective implementation that need to be addressed to maximize the impact of IE. Communication difficulties, particularly due to language differences and disruptive noise levels, can undermine the positive impact of IE. These can serve to hinder the formation of meaningful relationships and potentially compromise the wellbeing of older people. With careful planning, IE could contribute to the lives of older people resident in care facilities providing ongoing opportunities for personal growth, learning, and social engagement.

### 5.1 Design of intergenerational engagement

Our review found that IE programs varied in duration and frequency, with 6 weeks emerging as the most common length. The choice of a six-week duration for IE programs may reflect a compromise between realizing program goals and accommodating the schedules of diverse participants, allowing for structured engagement and outcome assessment. Though weekly sessions were the most common delivery model described (42, 43, 47–49, 51–53), session frequencies range from daily to monthly. Similarly, a systematic review by highlighted that meeting once a week in IE programs was found to be more beneficial for older people, with programs meeting more frequently than this showing decreased effectiveness (56).

The range of activities incorporated in the IE programs described in the included studies ranged from entertainment to educational, although all aimed at fostering meaningful exchange. Recent research suggests it is important to incorporate a range of activities into IE programs to cater to different interests and needs of participants. This variety means there is a greater chance that most needs will be accommodated (57). However, the research evaluating older people’s perceptions of the content of IE programs they received was limited as were opinions on optimum duration, frequency, and types of activity. This is consistent with the findings of a previous review (27), which highlighted shortcomings in evaluating components of IE programs in long-term care settings. Additionally, while the IE programs in our included studies featured a variety of activities, we were unable to compare the effects of different activities. Previous research suggests that the type of activity is less important than ensuring the experience is meaningful and has purpose for participants and is delivered in an environment that enables relationships to develop (58). This position pivots on the notion that engagement in IE (occupation) brings meaning and purpose to participants.

From our review we identified that the selection and design of activities in IE programs seemed to be selected on the age appropriateness of the CYP participants involved. We posit selection assumed that if the CYP felt confident or familiar with the activities the IE would contribute to facilitating relationship development. For example, adolescents and young adults were more often involved in IE involving more complex activities like reminiscence, creative art workshops, and learning sessions (43, 49, 51, 53, 54), leveraging their higher cognitive and communicative abilities to promote meaningful interactions with older people. Whereas the activities designed for IE involving preschool children participants involved simpler tasks like drawing and singing, aimed at fostering emotional connections rather than engagement in in-depth conversations (42, 44–48, 50). Adopting a planned approach to activity design underlines the necessity of tailoring programs to meet the capabilities of participants to ensure meaningful engagement ensues.

This resonates with an earlier review (18) that recommended IE programs should be tailored to the needs of users (CYP and older people) as this improved the effectiveness of the program. Another consideration is the diversity of older participants and sociocultural backgrounds, personal preferences, and any health conditions need to be accommodated when planning an IE program (59). Therefore, IE programs should be tailored to meet the specific needs and abilities of participants to ensure meaningful interactions that bring mutual benefits and foster enriching connections.

### 5.2 Measurement tools

Our review found an inconsistency in the measurement tools used across studies examining mental or emotional wellbeing among older people, making it difficult to undertake a meta-analysis. This issue underscores a critical need, also identified in prior reviews, for the development and use of standardized measurement tools in IE research (19, 27). Jarrott et al. suggested a range of tools that offer potential for broader scale comparability (57). Other researchers have pointed out the importance of developing a needs assessment and an outcome tool to better assess the effectiveness of future IE programs designed to enhance social connections for the growing older adult population (60). Developing and using standardized measurement tools for evaluating IE programs can lead to a more comprehensive understanding of what works, where, with whom, and in what setting. However, there are some challenges in ensuring that these tools measure consistently when applied to different populations and cultural settings. This makes it difficult to compare the effectiveness of IE programs across different settings, even though these tools provide some valuable insights. The design of new tools and adaptation of existing ones should take cultural and contextual factors into account, ensuring they are adapted for use in different cultural settings, without compromising their psychometric properties.

### 5.3 Enhancing mental and emotional well-being and reducing isolation

IE can foster emotional connections between older people and younger generations, crucial for enhancing mental health and emotional wellbeing. Our review found decreased levels of depression and loneliness among older people, highlighting the beneficial effects of IE on alleviating these affective symptoms. This adds to the growing body of research showing that IE programs can mitigate against loneliness and social exclusion and foster emotional well-being (61, 62). Furthermore, these programs have been consistently found to reduce depression in older participants (63–65). IE activities have the potential to narrow the generational divide, underscoring the value of social and emotional bonds (66). This suggests a significant role for IE in enhancing understanding and interaction across generations.

IE programs appear to foster connections and relationships between generations through facilitated interaction. This review indicates that the effect does not appear to be dependent on the age of the participants involved in the IE. For example, programs like the “Big and Mini,” created to link young adults with older adults through a custom website for weekly phone calls, illustrated how IE can mitigate risks associated with physical or mental health in later life by expanding older people’s support networks (67). IE appears to have potential for enhancing social networks and removing barriers to isolation.

### 5.4 Enriching older people’s lives through meaningful interaction

IE can play a role in enriching the lives of older people by facilitating meaningful connections with CYP. These interactions enhance social bonds and facilitate sharing of knowledge, experiences, and skills between generations. Newman & Hatton-Yeo found that IE programs enhance older people’s engagement with CYP by providing a window into modern education and lifestyles (68). This fosters mutual understanding, through sharing values, traditions, and technological knowledge, and providing personal fulfillment through community contribution for both generations (68). Participation in IE offers older people valuable learning experiences and positive interactions with CYP, which improves their understanding, motivation, and connection to society (69). IE seems to bring a sense of being valued, strengthen identity, and bring joy, thereby enriching the spiritual and cultural lives of older people and playing a role in improving their quality of life. This aligns with Teater’s findings, which show that IE can enhance older people’s sense of purpose, inclusivity, self-esteem, and overall life quality (70). These findings suggest that IE may provide older people with meaning and purpose, potentially enhancing their overall quality of life.

### 5.5 Cognitive stimulation in the context of intergenerational engagement

Our review found that IE may have a positive effect on improving memory in older people, although these changes are not always detected by traditional cognitive assessments like the MMSE. For example, qualitative feedback from older participants indicated perceived enhancements in memory and attention (52), while two included studies found that there were no significant changes in MMSE scores (48, 49). While subjective reports suggest improvements in some cognitive functions, objective measures may not fully capture these improvements. This aligns with a previous study where, despite no significant differences in MMSE scores between groups, the control group experienced a significant decline in hippocampal volume (an area crucial for memory) compared to the intervention group (71).

The MMSE is frequently used as a general test to measure cognitive impairment among older people, but it may not be sensitive enough to detect subtle changes in specific cognitive domains such as memory. Research shows that broader assessments may be required to measure the impact of cognitive domains. For instance, some researchers suggested that MMSE should be combined with additional tests to provide a fuller picture of older people’s cognitive function (72, 73). Moreover, other tools like BrainCheck have been recommended for detecting cognitive function by focusing on a range of cognitive skills rather than a general overview (74). Additionally, the integration of qualitative evidence can offer deeper insights into the experiences of older people, enriching our understanding of cognitive changes through IE intervention (52, 54). While the MMSE may not always detect subtle improvements in memory observed through IE, integrating qualitative insights and broader assessment tools can aid in gaining a comprehensive understanding of cognitive changes in older people within IE programs.

### 5.6 Navigating challenges to effective implementation of intergenerational engagement

The real-world contexts in which studies are delivered can produce unanticipated challenges for researchers. Identifying challenges encountered in previous studies to anticipate them and put in place mitigations will be pivotal to further IE study design. Our review identified language barriers and noisy environments as potential challenges that could reduce the benefits of IE (46, 51, 52). Creating the right environment conducive to intergenerational interaction, by reducing noise, considering the acoustic aspects of venues in advance, and implementing language support services may help prevent problems. Training long-term care staff in effective communication and facilitation techniques may ensure staff are equipped to support and enhance IE interactions if adopted more widely. Those implementing IE programs should ensure both older people and CYP are adequately prepared for their engagement (75). This preparation might include the provision of health and social support, visual and hearing assistance, comfortable environments, and the IE schedule tailored to accommodate the daily routines of older participants. With careful planning, these challenges can be effectively addressed to ensure successful implementation of IE programs.

### 5.7 Implications for future research and practice

Only three studies included in this review utilized mixed methods designs (45, 46, 51). Future IE research employing mixed method designs may more comprehensively provide evidence on the impacts and participant experiences of IE programs. Another limitation of all the studies included in this review is the absence of anxiety assessments. Anxiety commonly experienced alongside depression and frequently measured in conjunction, is reported as prevalent among older people living in long-term care facilities (76, 77). Future IE research should incorporate assessments of anxiety to offer a more comprehensive understanding of IE’s potential impact on mental health among older people living in long-term care settings. This review included studies from mainland China, Taiwan, South Korea, Hong Kong, Singapore, and Japan. The four studies from mainland China included three studies of IE involving preschool children (42, 44, 45) and one with young adults (54). Notably, there exists a gap in IE research concerning the involvement of school children and adolescents in the context of mainland China, where age ranges from 6 to 17 years old (78).

Tailoring IE programs to the specific needs and abilities of participants is crucial for their effectiveness. In this case involving older people resident in long term care, their caregivers, the children/young people who may be participants in the delivery of an IE program, their teachers and parents, might improve program efficacy and cost-effectiveness (79–81). While there is interest in using IE to enhance the health and well-being of older individuals in long-term care facilities, none of the studies included in this review demonstrated the involvement of stakeholders in program development or research design. Additionally, the MMAT did not consider stakeholder involvement as a criterion for assessing study quality. Involving the public and patients in research, known as public and patient involvement and engagement (PPIE), improves research through their participation in various aspects, such as design, conduct, and dissemination of studies (82). This enables the findings to reach wider audiences so they can have a greater impact (82). It is more likely to ensure research and its outcomes (high quality care and treatment) has any impact, by working with those who experience the problem, need, or use the services under investigation, or those who provide the care, design the services, or people who make decisions on the resource allocation, or other stakeholders. Integrating PPIE into research and intervention design, especially in IE programs, has great potential to improve effectiveness and relevance to stakeholders, ultimately making interventions more meaningful. Previous researchers (57) have suggested that engagement with stakeholders of IE warrants further exploration so the link between best practice and outcomes is increased. This might enhance estimation of the dose (e.g., length, frequency), content, measurement, and quality of IE programs. Adopting a more participatory co-designed approach (83) holds promise and may ultimately maximize the humanizing potential of IE, improve the experience, and bring mutual benefits for both older and CYP participants.

For effective implementation of IE programs, recognizing and addressing any barriers is important. Our review suggests that language differences between CYP and older people participants and the environments where IE took place reduced the effectiveness of IE programs. In linguistically diverse contexts such as mainland China (84, 85), effectively addressing language barriers is crucial for the successful implementation of IE programs. Incorporating activities such as art and music workshops, and language learning where older people teach local dialects and the younger generation share their knowledge of Mandarin for example may help to bridge these gaps. To minimize adverse auditory effects that could impact the IE experience, it is important to carefully choose the right environment. This selection process should include evaluating the size of the space relative to the number of participants, considering floor coverings to reduce noise, addressing extraneous sounds like air conditioning, accommodating any special needs of those with hearing impairments, and preparing and preparing CYP participants for better interaction with older participants. Aligning scheduling of IE programs with the daily routines in facilities will also minimize disruptions, ensuring IE programs can be incorporated more easily in the daily routines of participating older people and/or others residing in the facilities.

### 5.8 Strengths and limitations

This systematic review assessed the effectiveness and experiences of IE among older people in long-term care facilities in Asia, providing a detailed analysis of its impacts. By including studies published in English or Chinese from 2000 to the present, the review seeks to understand both historical and current IE practices and perspectives. Its focus on Asian long-term care facilities highlights culturally specific practices that could contribute to future research design in this area.

Inevitably any review has limitations. Restricting the review to published studies in English or Chinese may have resulted in the exclusion of relevant studies published in other languages. This is particularly significant given the diversity of languages across Asia. By limiting the inclusion criteria to empirical studies, the review may have overlooked valuable insights from secondary research, gray literature, expert opinions, policy, and theoretical analyses that could contribute to a deeper understanding of the potential or impacts of IE. Lastly, the temporal restriction to studies published from 2000 onwards, while aiming to ensure relevance, capture trends may have excluded historical perspectives that could potentially provide valuable context for understanding the evolution of IE practices in Asia.

## 6 Conclusion

This review suggests that IE could be beneficial in reducing depression and loneliness, enhancing the quality of life, and strengthening social bonds for older people living in Asian long-term care facilities. Despite IE programs’ variability and some challenges associated with implementation, the evidence supports the adoption of IE as a strategy to address the emotional and social needs of older people in long-term care facilities. Future research should focus on refining intervention, study designs and overcoming other challenges to successful implementation of IE in Asian long-term care facilities. We would recommend involvement of stakeholders in the development of any future intervention and research design to enhance transparency, public accountability, and alignment with needs of the populations our research should benefit.

## Data availability statement

The original contributions presented in the study are included in the article/Supplementary material, further inquiries can be directed to the corresponding author.

## Author contributions

HL: Conceptualization, Data curation, Formal analysis, Investigation, Methodology, Project administration, Writing – original draft, Writing – review & editing. AT: Conceptualization, Supervision, Validation, Writing – review & editing, Methodology. PG: Conceptualization, Methodology, Supervision, Validation, Writing – review & editing.
