# Supplementary material for: Intergenerational engagement with Asian residents in long-term care facilities: a mixed method systematic review
Source: Front Public Health. 2024 Jul 16;12:1422134. doi: 10.3389/fpubh.2024.1422134 (PMC11286590; doi:10.3389/fpubh.2024.1422134)
Supplement: Supplementary file 1 [file Data_Sheet_1.PDF]

Supplementary Table 1. Study quality assessment

	Li et al.(2022)	Kim & Lee (2018)	Wang (2023)	Wang & Wang (2022)	LinOu (2004)	Hong & Yao (2017)	Tsai & Lin (2022)	Chung (2009)	Leong et al. (2021)	Morita & Kobayashi (2013)	Hwang et al. (2014)	Fan (2010)	Wang (2020)
**Screening questions**													
1. Are there clear research questions?	✓	✓	✗	✓	✓	✓	✓	✓	✓	✓	✓	✓	✓
2. Do the collected data allow to address the research questions?	✓	✓	✓	✓	✓	✗	✓	✓	✓	✓	✓	✓	✓
**1.Qualitative**													
1.1. Is the qualitative approach appropriate to answer the research question?				✓	✓				✓		✓	✓	✓
1.2. Are the qualitative data collection methods adequate to address the research question?				✓	✓				✓		✓	✓	✓
1.3. Are the findings adequately derived from the data?				✓	✗				✓		?	✓	✓
1.4. Is the interpretation of results sufficiently substantiated by data?				✓	✗				✓		✗	✓	✓
1.5. Is there coherence between qualitative data sources, collection, analysis and interpretation?				✓	✗				✓		✗	✓	✓
**2.Quantitative, RCT**													
2.1. Is randomization appropriately performed?			✓										
2.2. Are the groups comparable at baseline?			✓										
2.3. Are there complete outcome data?			?										
2.4. Are outcome assessors blinded to the intervention provided?			✗										
2.5 Did the participants adhere to the assigned intervention?			✓										
**3.Quantitative, non-randomized**													
3.1. Are the participants representative of the target population?	✓	✓		✗	✗	?	?	?			?		
3.2. Are measurements appropriate regarding both the outcome and intervention (or exposure)?	✓	✓		✓	✓	?	✓	✓			✓		
3.3. Are there complete outcome data?	✓	✓		✓	✓	✓	✓	✓			✓		
3.4. Are the confounders accounted for in the design and analysis?	?	✓		✓	✗	?	✓	?			?		
3.5. During the study period, is the intervention administered (or exposure occurred) as intended?	?	✓		✓	✓	✓	✓	✓			✓		
**4. Quantitative descriptive**													
4.1. Is sampling strategy relevant to address research question?										✓			
4.2. Is the sample representative of the population under study?										✗			
4.3. Are measurements appropriate?										✓			
4.4. Is the risk of nonresponse bias low?										?			
4.5. Is the statistical analysis appropriate to answer the research question?										✓			
**5.Mixed methods**													
5.1. Is there an adequate rationale for using a mixed methods design to address the research question?				✓	✓						✓		
5.2. Are the different components of the study effectively integrated to answer the research question?				✓	?						✗		
5.3. Are the outputs of the integration of qualitative and quantitative components adequately interpreted?				✓	?						✗		
5.4. Are divergences and inconsistencies between quantitative and qualitative results adequately addressed?				✓	✗						✗		
5.5. Do the different components of the study adhere to the quality criteria of each tradition of the methods involved?				✓	✗						✗		

Yes= ✓ No = ✗ can't tell= ?
